# A Cost-Effective Two-Step Approach for Multi-Cancer Early Detection in High-Risk Populations

**DOI:** 10.1158/2767-9764.CRC-24-0508

**Published:** 2025-01-24

**Authors:** Shuaipeng Geng, Shiyong Li, Wei Wu, Yinyin Chang, Mao Mao

**Affiliations:** 1 Clinical Laboratories, Shenyou Bio, Zhengzhou, China.; 2 Research & Development, SeekIn Inc, Shenzhen, China.; 3 Research & Development, SeekIn Inc, San Diego, California.; 4 Yonsei Song-Dang Institute for Cancer Research, Yonsei University, Seoul, Republic of Korea.

## Abstract

In population-wide cancer screening, three key issues need to be focused on: the number of cancer cases identified, the number of false positives, and the cost. OncoSeek is a multi-cancer early detection (MCED) test using seven protein tumor markers and artificial intelligence. SeekInCare is an MCED test that integrates the seven protein tumor markers and four cancer genomic features from cell-free DNA by shallow whole-genome sequencing. In a two-step approach, the initial screening is conducted using OncoSeek, and SeekInCare is then used as the secondary test for individuals who tested positive by OncoSeek. We simulated a screening in five million adults ages ≥50 years with a cancer incidence rate of 1.9%. Whereas at 91.0% specificity OncoSeek had 441,450 false positives, using the two-step approach significantly reduced false positives to 34,335 (0.7%). Although SeekInCare and Galleri identified more cancer cases (32,015 and 27,455, respectively) than the two-step MCED (21,280), their total costs reached $3,750 million and $4,745 million, respectively. As the positive predictive value of two-step MCED (38.3%) was comparable with SeekInCare (27.7%) and Galleri (38.3%), it reduced the cost by 5.3-fold and 6.6-fold, respectively, amounting to a total cost of $713.6 million and a cost of $143 per individual screened. The cost of per cancer case detected was $117,133 for SeekInCare and $172,828 for Galleri, which were 3.5-fold and 5.2-fold higher, respectively, than the two-step MCED ($33,534). The two-step approach not only significantly reduces false positives but also cuts down the screening cost substantially, making it a cost-effective strategy for population-wide cancer screening.

**Significance:**

Large-scale screening inevitably leads to significant financial burdens on the healthcare system, which is a key factor constraining nationwide screenings. The two-step MCED approach not only maintains comparable performance but also substantially alleviates financial strains compared with the direct use of next-generation sequencing–based MCED tests for massive screenings.

## Introduction

In population-wide cancer screening, three key aspects need to be focused on: the number of cancer cases identified, the number of false positives, and the cost of screening. A multi-cancer early detection (MCED) test is likely more effective than a single-cancer early detection test in a single screening session, given its capability to detect multiple cancer types at once (1). Multiple single-cancer early detection tests for screening multiple cancer types can have higher false positive rates as the false positives are accumulative from different tests, whereas an MCED test simultaneously targets multiple cancer types with a single, lower false positive rate (2, 3). A high false positive rate not only results in a significant waste of healthcare resources but also imposes unnecessary psychologic and financial burdens on individuals. Additionally, the high cost of screening can overburden the public healthcare system and impede the implementation of widespread screening programs. Large-scale screening inevitably leads to significant financial burdens on the healthcare system, which is a key factor constraining nationwide screenings, even in high-income countries. This challenge is exacerbated by the introduction of state-of-the-art MCED tests based on next-generation sequencing (NGS), even when the cost of sequencing will decrease in the coming years (1).

To date, there are well-established screenings for several cancer types, including breast, cervical, colon, lung, oral, prostate, skin, and stomach cancers. The screenings for prostate and colon cancers typically include two steps to avoid financial burdens on individuals and the public healthcare system. Prostate cancer screening starts with a PSA blood test and then uses an MRI to rule out false positives (4). Similarly, colon cancer screening starts with a stool-based fecal immunochemical test and then uses colonoscopy to verify positive results (5). This two-step approach is much more cost-effective than initiating with costly and resource-intensive MRI and colonoscopy. This approach has been implemented worldwide, especially in countries with universal health coverage such as the United Kingdom and the Netherlands.

In this study, we propose a two-step MCED screening approach using low-cost screening tests (such as OncoSeek) for the initial screening, followed by more superior screening tests (such as SeekInCare and Galleri) as the secondary test for individuals who tested positive in the initial screening. This two-step approach can significantly reduce the financial burden compared with using NGS-based MCED tests for population-level screening.

## Materials and Methods

### Study design

OncoSeek is a test for MCED using a panel of seven selected blood-based protein tumor markers (PTM) that was quantified by common clinical electrochemiluminescence immunoassay analyzers (6). The SeekInCare test, an advanced version of OncoSeek, further enhances MCED by integrating OncoSeek results with cancer-specific genomic features—copy-number aberration (CNA), fragment size (FS), end motifs, and oncogenic viruses—from circulating-free DNA (cfDNA) using shallow whole-genome sequencing (sWGS) at 3× coverage (7, 8). In our proposed two-step MCED approach, OncoSeek is initially used for primary screening, providing a probability of cancer (POC) index. Then SeekInCare is used for a second round of testing on the positive results with a POC index of 0.5 or higher, which generates a cancer risk score (CRS) to further evaluate the cancer risk. In the second-round test, the PTMs used by SeekInCare can carry over the results from OncoSeek in the primary screening, thereby avoiding the costs associated with retesting the PTMs. OncoSeek and SeekInCare received the conformité européene - in vitro diagnostic (CE-IVD) mark (i.e., the regulatory approval in the European Union) in 2022.

### Study participants

Newly diagnosed patients with cancer (*n* = 617) who were admitted to five hospitals (Peking University Shenzhen Hospital, Sun Yat-sen Memorial Hospital, Sun Yat-sen University Cancer Center, The First Affiliated Hospital of Zhengzhou University, and Henan Cancer Hospital) were enrolled in this study. Noncancer individuals (*n* = 580) without cancer history or cancer-related symptoms were selected from the physical examination center of Sun Yat-sen Memorial Hospital as the control group. In addition, our employees and their parents were also included as noncancer controls. The study was approved by the independent ethics committee of the leading site, Peking University Shenzhen Hospital (No. 2019.052). Inclusion criteria for cancer patients included confirmed pathology diagnoses and no prior antitumor treatments. Noncancer individuals were excluded if they had a malignancy presence or history or if they had undergone organ transplantation or were pregnant. All participants provided written informed consent upon enrollment. A measure of 8 mL of peripheral blood from each participant was collected using a Cell-Free DNA BCT blood collection tube (Streck) after enrollment. All patients with cancer were treatment-naïve at the time of blood draw. For further details on the cohort, refer to previous publications (6–9).

### OncoSeek algorithm for PTM analysis

Refer to our prior publication for the analysis of seven selected PTMs (AFP, CA125, CA15-3, CA19-9, CA72-4, CEA, and CYFRA 21-1; ref. 6) In brief, Roche cobas e411 (Roche Diagnostics GmbH) was utilized to measure the concentration of seven PTMs in 500 μL plasma samples following the manufacturer’s instructions. By integrating and leveraging the performance of all seven PTMs, an algorithm named OncoSeek was constructed based on the generalized linear model method.

### Multi-omics SeekInCare analysis and weighted CRS algorithm

See the previous publications for plasma sample preparation, DNA extraction, sequencing library construction, and sWGS (7–9). In brief, cfDNA was extracted from plasma using QIAamp Circulating Nucleic Acid Kit (QIAGEN) following the manufacturer’s instructions. The isolated cfDNA was utilized for library construction using KAPA HyperPrep Kit (Kapa Biosystems), adhering to the provided protocol. These prepared libraries were then subjected to sWGS (∼3× coverage) on X Ten or the NovaSeq platform (Illumina).

Sequence alignment, cfDNA CNA detection, end motif analysis, and oncogenic viral DNA analyses were described in previous publications (7–9). We used the global FS short ratio P150, detailed in our previous studies (7, 9), along with the 5-Mb regional short-to-long ratio features, described in the publication (10), to calculate the FS score using the gradient-boosting machine machine-learning method.

Through the SeekInCare test, the genomic features (CNA, FS, end motif, and oncogenic virus) were analyzed by the sWGS data. Additionally, the PTM value from OncoSeek was incorporated as an additional feature. We used a linear regression approach to integrate the value of the five features to calculate the CRS with the following formula: CRS = a × CNA + b × FS + c × end motif + d × virus + e × PTMs, in which a, b, c, d, and e represent the weight values of CNA, FS, end motif, virus, and PTMs, respectively, which were determined by grid search to get the highest area under the ROC curve (AUC; ref. 11). Finally, the CRS cutoff at 98% specificity was set at 2.0; a score of CRS ≥2.0 classifies a case as cancer.

### Statistical analysis

All statistical analyses were performed using R (v3.5.3, RRID: SCR_001905). pROC (v1.13.0, RRID: SCR_024286) was used to estimate the performance of ROC for classifying patients with cancer and noncancer subjects. Sensitivity, specificity, and predictive values were calculated with the epiR (v0.9-99, RRID: SCR_021673) package.

### Data availability

All data generated or analyzed during this study are included in this article, its supplementary information files, and publicly available repositories. Sequencing data of the patients with cancer and noncancer individuals, as well as clinical information about these patients, are available with controlled access at https://ngdc.cncb.ac.cn with accession numbers PRJCA017828, PRJCA022396, and PRJCA022679.

## Results

### Participant demographics

A total of 1,197 participants were enrolled in this case–control study. The cancer group (*n* = 617) and noncancer group (*n* = 580) were nearly comparable in terms of gender (χ^2^ test, *P* = 0.05). The average age of patients with cancer was higher at 56.0 years compared with the noncancer group, which had an average age of 48.3 years (Table 1). Among the patients with cancer, majority (66.3%) exhibited localized disease (stage I: 26.3%; stage II: 18.6%; and stage III: 21.4%). There were 6.2% of patients with missing tumor stages and 5.3% of patients (e.g., leukemia) who were inapplicable for the American Joint Committee on Cancer Staging Manual (8th edition; ref. 12). A total of 27 cancer types were included in this case–control study (Supplementary Table S1).

**Table 1:** Case–control cohort demographic

	Cancer (*n* = 617)	Noncancer (*n* = 580)
Age, years		
Mean (SD)	56.0 (13.7)	48.3 (12.9)
Age group, *n* (%)		
<50 years	179 (29.0)	303 (52.2)
≥50 years	432 (70.0)	257 (44.3)
Missing	6 (1.0)	20 (3.4)
Sex, *n* (%)		
Male	351 (56.9)	296 (51.0)
Female	266 (43.1)	284 (49.0)
Stage, *n* (%)		
I	162 (26.3)	
II	115 (18.6)	
III	132 (21.4)	
IV	137 (22.2)	
Not expected to be staged	33 (5.3)	
Missing	38 (6.2)	

### OncoSeek and SeekInCare performances in the case–control study

**Figure 1:**
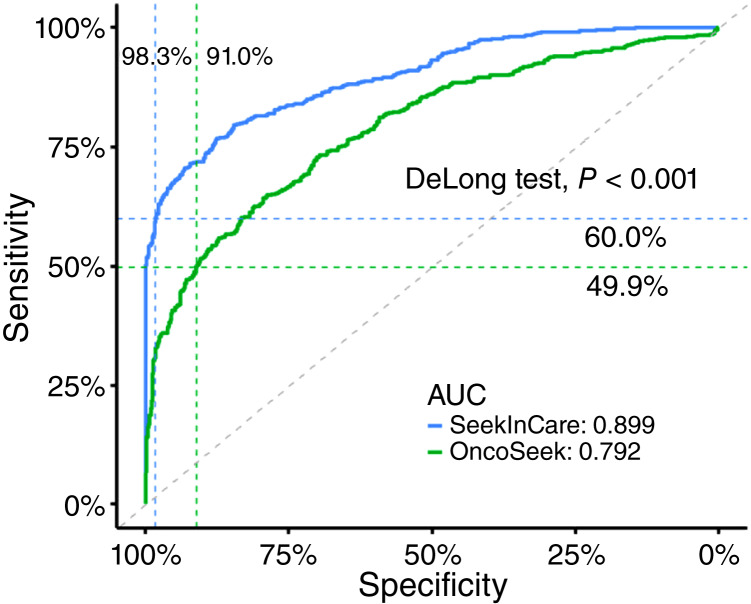
ROC curves of OncoSeek and SeekInCare in the case–control study. The area under the ROC curves (AUCs) of OncoSeek and SeekInCare were 0.792 and 0.899, respectively, showing a significant difference (*P* < 0.001 by the DeLong test).

All 1,197 participants from this case–control study with both OncoSeek and SeekInCare results were included. OncoSeek and SeekInCare could distinguish patients with cancer from noncancer individuals with AUCs of 0.792 and 0.899, respectively (*P* < 0.001 by the DeLong test; Fig. 1). Among the genomic features in SeekInCare, the AUCs for CNA, FS, end motif, and oncogenic virus were 0.797, 0.865, 0.877, and 0.654, respectively (Supplementary Fig. S1). With a POC 0.5 as the cutoff for OncoSeek and a CRS 2.0 as the cutoff for SeekInCare, OncoSeek showed a sensitivity of 49.9% at 91.0% specificity, whereas SeekInCare demonstrated a sensitivity of 60.0% at 98.3% specificity (Fig. 1; Table 2). In GRAIL’s circulating cell-free genome atlas (CCGA) study (13), a similar case–control MCED study, involving 2,823 patients with cancer and 1,254 noncancer individuals, GRAIL’s Galleri test demonstrated a sensitivity of 51.5% at 99.5% specificity (Table 2).

**Table 2:** Sensitivity and specificity in case–control studies: OncoSeek, SeekInCare, two-step MCED, and Galleri

	OncoSeek	SeekInCare	Two-step MCED	Galleri^a^
Sensitivity	49.9%	60.0%	39.9%	51.5%
Specificity	91.0%	98.3%	99.3%	99.5%

a The sensitivity and specificity of Galleri were based on GRAIL’s CCGA study.

### Sensitivity and specificity of the two-step approach

**Figure 2:**
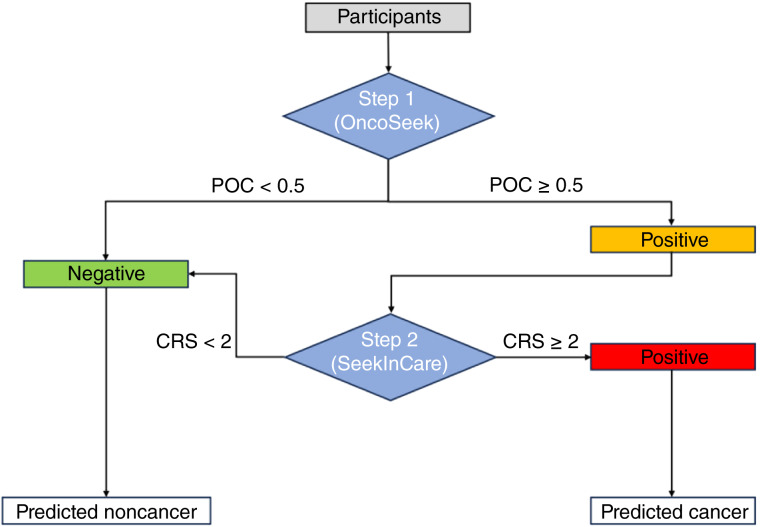
Two-step MCED approach flowchart. Participants first underwent initial testing with OncoSeek. If POC ≥0.5, the individuals were classified as initially positive and proceeded to secondary testing with SeekInCare. If POC <0.5, the individuals were classified as negative and predicted as noncancer. In secondary testing, if CRS ≥2, the initial positives were classified as secondary positives and predicted as cancer. If CRS <2, the initial positives were considered negative and predicted as noncancer.

**Figure 3:**
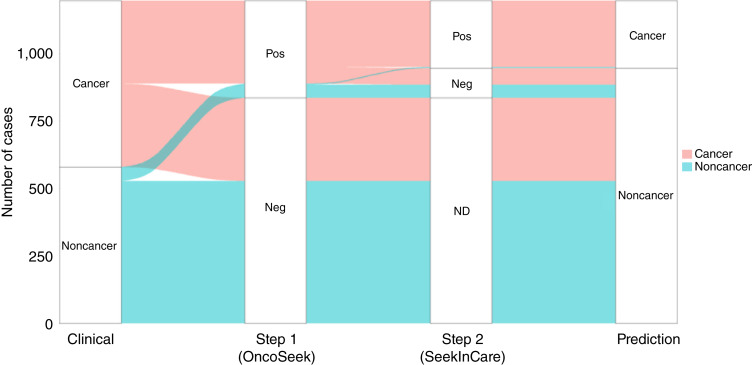
Outcome of the two-step MCED approach in the case–control study. In the case–control cohort, the two-step MCED approach significantly improved specificity from 91.0% to 99.3%, whereas sensitivity decreased from 49.9% to 39.9%. ND, no detection; Neg, negative; Pos, positive.

When we conducted SeekInCare as a secondary test on the individuals identified as positive (POC ≥0.5) based on the initial testing results from OncoSeek (Fig. 2) in the case–control cohort, the specificity significantly increased from 91.0% to 99.3%, whereas the sensitivity decreased from 49.9% to 39.9% (Fig. 3). Therefore, the false positive rate was dramatically reduced, lowering 12.9-fold from 9.0% to 0.7% after the secondary testing with SeekInCare. Supplementary Table S2 presented the sensitivities and specificities of OncoSeek and the two-step approach across different cancer stages and types.

### Cost simulating of the two-step approach and Galleri

**Figure 4:**
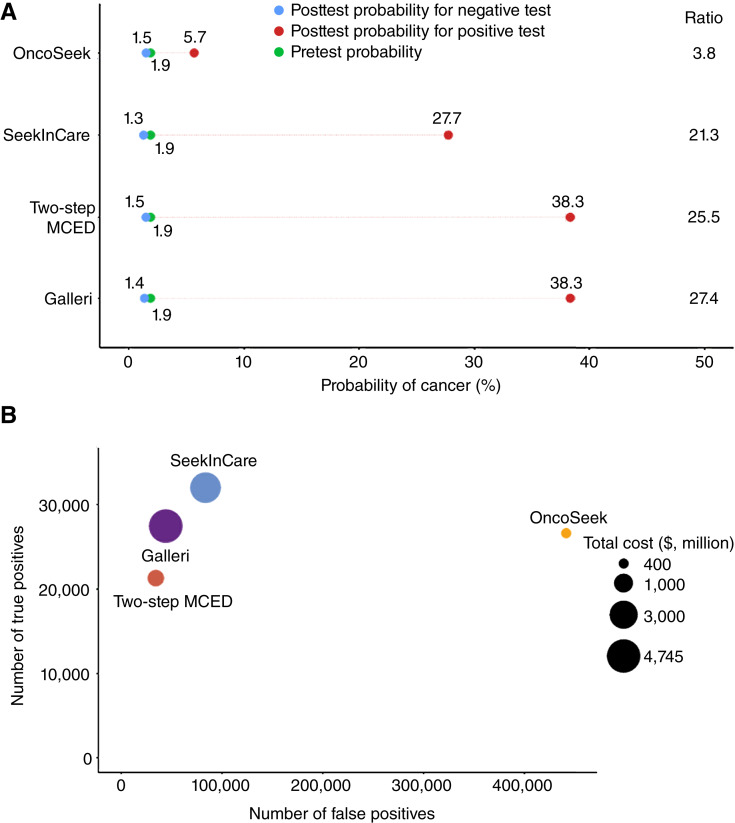
Performance and cost-effectiveness comparison in a screening of five million people. **A,** Pretest probability and posttest probability for cancer with positive and negative results. Ratio, the ratio of posttest probability for positive to negative. **B,** Detailed breakdown of the cost implications and detection probabilities for each approach.

We then simulated conducting MCED screening in a population of five million adults, ages 50 years or older, using the same inclusion criteria as GRAIL’s PATHFINDER study (2). This demographic represents a high-risk population with a relatively high incidence of cancer, such as the eligible population for MCED screening in the United States. The parameters used aligned with those from the PATHFINDER study, and we accordingly simulated a cancer incidence rate of 1.9%. Due to the unavailable real-world data, the simulated real-world sensitivities of OncoSeek, SeekInCare, and the two-step approach were adjusted downward proportionally to 28.0%, 33.7%, and 22.4%, respectively, based on the sensitivity differences observed between GRAIL’s CCGA study (51.5% sensitivity in a retrospective setting) and PATHFINDER study (28.9% sensitivity in a prospective setting; Table 3). We did not adjust specificity as the specificities in CCGA, PATHFINDER, and the two-step case–control study were very similar, with 99.5%, 99.1%, and 99.3%, respectively. The comparison of the efficiency among the four screening approaches in the simulated screening is shown in Fig. 4A. We found that despite the specificity of OncoSeek being as high as 91.0%, in a large-scale screening of five million individuals, there were 441,450 cases of false positives (9.0%). Using the two-step MCED approach could significantly reduce the number of false positives to 34,335 (0.7%) for the OncoSeek positive individuals, which was even lower than that of the superior NGS-based MCED tests, SeekInCare and Galleri, with false positives of 83,385 (1.7%) and 44,145 (0.9%), respectively (Fig. 4B).

**Table 3:** Simulated performance, efficiency, and cost in a screening of five million people

	OncoSeek	SeekInCare	Two-step MCED	Galleri
Performance and efficiency^a^				
Sensitivity^b^	28.0%	33.7%	22.4%	28.9%
Specificity	91.0%	98.3%	99.3%	99.1%
Number of true positives	26,600	32,015	21,280	27,455
Number of false positives	441,450	83,385	34,335	44,145
Number of true negatives	4,463,550	4,821,615	4,870,665	4,860,855
Number of false negatives	68,400	62,985	73,720	67,545
PPV	5.7%	27.7%	38.3%	38.3%
Negative predictive value	98.5%	98.7%	98.5%	98.6%
Positive likelihood ratio	3.1	19.8	32.0	32.1
Negative likelihood ratio	0.8	0.7	0.8	0.7
Pretest probability	1.9%	1.9%	1.9%	1.9%
Posttest probability for positive test	5.7%	27.7%	38.3%	38.3%
Posttest probability for negative test	1.5%	1.3%	1.5%	1.4%
NNS^c^	188	156	235	182
Cost				
Total	$400 million	$3,750 million	$713.6 million	$4,745 million
Per patient with cancer identified	$15,038	$117,133	$33,534	$172,828
Per individual screened	$80	$750	$143	$949

a The simulated real-world cancer incidence rate was based on GRAIL’s PATHFINDER study.

b The simulated real-world sensitivity was adjusted downward proportionally based on the sensitivity differences observed between the CCGA study (case–control) and the PATHFINDER study (prospective).

c NNS is defined as the number needed to be screened for the detection of one cancer case.

Furthermore, in terms of screening costs, although SeekInCare and Galleri identified more cancer cases (32,015 and 27,455, respectively) than the two-step MCED approach (21,280), their total costs were significantly high, reaching $3,750 million and $4,745 million respectively, equating to $750 for SeekInCare (based on the assumptions of reagent/consumable cost, instrument depreciation, labor cost, tax, profit margin, etc.) and $949 for Galleri per individual screened (Fig. 4B).

Whereas the positive predictive value (PPV) and the number needed to screen (NNS) for the detection of one cancer case of the two-step MCED approach (PPV, 38.3%; NNS, 235) were comparable with SeekInCare (PPV, 27.7%; NNS, 156) and Galleri (PPV, 38.3%; NNS, 182), it reduced the cost significantly (i.e., 5.3-fold and 6.6-fold reductions, respectively), amounting to a total cost of $713.6 million and cost per individual screened of $143 ($80 per test for OncoSeek and $670 additional cost per test for SeekInCare; Table 3). The cost of per cancer case detected using the two-step MCED approach was $33,534, which was substantially less than the costs of SeekInCare and Galleri, which were 3.5 times and 5.2 times higher, amounting to $117,133 and $172,828, respectively (Table 3). Hence, the two-step approach is more cost-effective than using superior screening tests like SeekInCare and Galleri alone.

## Discussion

Screening for cancer types like breast, cervical, colon, lung, oral, prostate, skin, and stomach cancers is recommended for asymptomatic individuals based on test availability and cancer prevalence in various countries. In the United States, the United States Preventive Services Task Force recommends screening four cancer types, namely, breast, cervical, colon, and lung cancers, which account for approximately 25% of all cancers and may detect nearly 15% of all cancer cases (14, 15). Therefore, the upcoming blood-based MCED tests that detect cancer DNA and protein in the bloodstream have the potential to detect the remaining 75% of cancers not covered by the current screenings, as well as the cancer cases missed by the current screenings.

New screening tests inevitably place additional costs on the healthcare system. This is especially true with the introduction of the state-of-the-art MCED tests that are based on NGS. For example, Galleri is $949 per test, and SeekInCare is $750. SeekInCare leverages PTM results from OncoSeek as the secondary test, effectively eliminating the PTM retesting costs and reducing the overall cost to $670. Healthcare expenditure in both developed and developing countries has continued to grow over the recent decades. This is due to both significant medical advances as well as the spending related to aging. The challenge for all countries is to enhance health systems by improving healthcare quality and accessibility while controlling expenditure growth. According to a recent study, the United States spent $43 billion on screening in 2021 to detect five cancers (breast, cervical, colorectal, lung, and prostate; ref. 16). The largest driver of the expenditure was colonoscopy, which accounted for 55% of the total screening cost. The high cost of colon cancer screening is largely driven by colonoscopy being used as a primary screening for colon cancer in the United States instead of the two-step approach (fecal immunochemical test followed by colonoscopy) used in countries with universal health coverage, such as the United Kingdom and the Netherlands.

In this study, we started with assessing the performances of a low-cost MCED test (OncoSeek) and a high-cost MCED test (SeekInCare) in a case–control study and then demonstrated that the combined approach of the two tests (i.e., two-step MCED) would maintain comparable performance with NGS-based MCED tests (SeekInCare and Galleri). By simulating the two-step approach and high-cost/high-performance MCED tests (SeekInCare and Galleri) in a screening of five million people, the two-step approach demonstrated that the cost of per cancer case detected was 3.5 times and 5.2 times less than that of SeekInCare and Galleri, respectively, whereas the number of cancer cases identified was reduced by only 33.5% and 22.5%.

We would like to emphasize here that in resource-constrained settings, the actual priority is not a high specificity but a high sensitivity. If we miss detecting a cancer in such a population which does not usually seek medical advice until the cancer is at an advanced stage, it results in a loss of opportunity. The individual will be wrongly reassured, the cancer will progress, and the diagnosis will come too late. Therefore, in places such as low-income countries, OncoSeek would be the sole screening test. OncoSeek can also be effectively used as an auxiliary diagnostic tool for individuals with cancer-related symptoms in low-resource settings as it has sufficient sensitivity (52%) and accurate tissue of origin prediction (67%) across nine common cancer types (6).

As this study relies primarily on the simulations drawn from retrospective data and a simulated MCED screening cohort rather than real-world data, a prospective study in the real-world setting is needed to provide stronger evidence supporting the two-step approach for population-level screening. The healthcare expenditure over quality-adjusted life year gain should be simulated using the key parameters in this study and the cost of standard-of-care therapy for each common cancer type in the countries like the United States to address the cost-effectiveness of the two-step MCED approach.

In conclusion, the two-step MCED approach not only significantly reduces the false positive rate but also helps cut down a significant amount of screening cost, making it a cost-effective strategy for nationwide screening.

## Supplementary Material

Figure S1, Table S1, Table S2 Supplementary Data
