# Supplementary material for: A Cost-Effective Two-Step Approach for Multi-Cancer Early Detection in High-Risk Populations
Source: Cancer Res Commun. 2025 Jan 24;5(1):150–6. doi: 10.1158/2767-9764.CRC-24-0508 (PMC11758400; doi:10.1158/2767-9764.CRC-24-0508)
Supplement: Figure S1, Table S1, Table S2 — Supplementary Data [file crc-24-0508_figure_s1_table_s1_table_s2_suppsf1-st1-st2.doc]

**Supplementary Materials**

**A cost-effective two-step approach for multi-cancer early detection in high-risk populations**

Shuaipeng Geng^1,#^, Shiyong Li^2,#^, Wei Wu^2^, Yinyin Chang^1^, Mao Mao^3,4,*^

**Affiliations:**

^1^Clinical Laboratories, Shenyou Bio, Zhengzhou 450000, China

^2^Research & Development, SeekIn Inc, Shenzhen 518000, China

^3^Research & Development, SeekIn Inc, San Diego 92121, USA

^4^Yonsei Song-Dang Institute for Cancer Research, Yonsei University, Seoul 03722, Republic of Korea

^#^Authors contributed equally

^*^Corresponding author

Corresponding author: Mao Mao, 10320 Camino Santa Fe, Suite G, San Diego, CA 92121, USA; E-mail address: maomao@yuhs.ac. ORCID: 0000-0002-8570-8571.

**Supplementary Figures**


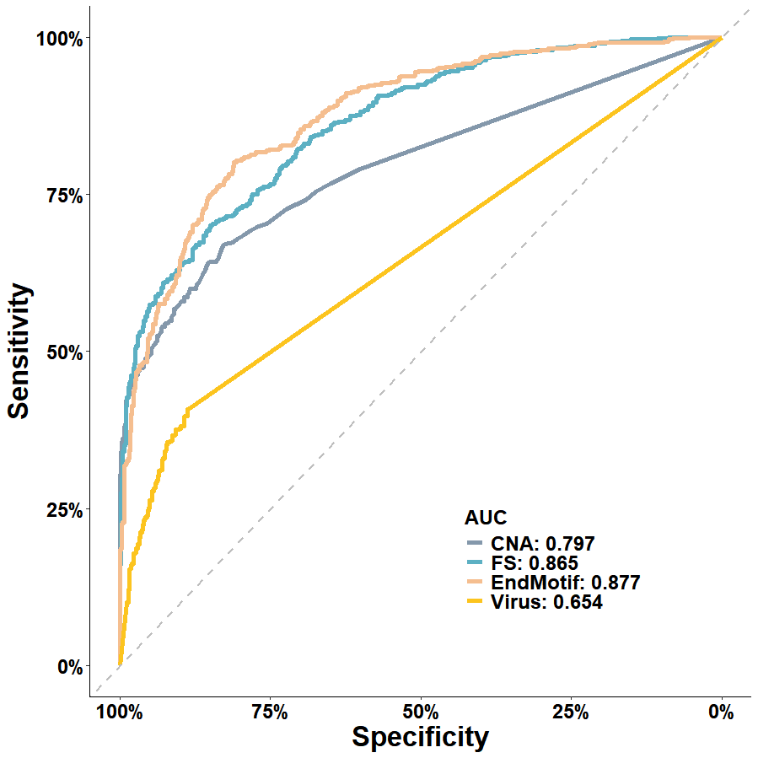


**Figure S1 Performance of the genomic features in MCED.** Among the genomic features in SeekInCare for MCED, the AUCs for CNA, FS, EndMotif, and oncogenic virus were 0.797, 0.865, 0.877, and 0.654, respectively. AUC, area under curve.

**Supplementary Tables**

**Supplementary Table 1 The proportion of each cancer type in the** case-control study

| **Cancer Type** | **Sample (n)** | **Percentage (%)** |
| --- | --- | --- |
| Lymphoma | 146 | 23.7 |
| Liver | 129 | 20.9 |
| Colorectum | 62 | 10.0 |
| Stomach | 56 | 9.1 |
| Breast | 52 | 8.4 |
| Lung | 43 | 7.0 |
| Leukemia | 30 | 4.9 |
| Pancreas | 17 | 2.8 |
| Esophagus | 10 | 1.6 |
| Gallbladder | 10 | 1.6 |
| Bile Duct | 9 | 1.5 |
| Cervix | 8 | 1.3 |
| Thyroid | 8 | 1.3 |
| Bladder | 5 | 0.8 |
| Ovary | 5 | 0.8 |
| Renal | 4 | 0.6 |
| Duodenal | 3 | 0.5 |
| Endometrial | 3 | 0.5 |
| Multiple Myeloma | 3 | 0.5 |
| Prostate | 3 | 0.5 |
| Nasopharyngeal | 2 | 0.3 |
| Gastrointestinal stromal tumor | 1 | 0.2 |
| Glioblastoma | 1 | 0.2 |
| Glottic | 1 | 0.2 |
| Oropharyngeal | 1 | 0.2 |
| Periampullary | 1 | 0.2 |
| Ureteral | 1 | 0.2 |
| Unknown primary | 3 | 0.5 |

**Supplementary Table 2 Comparison of the sensitivities and specificities of OncoSeek, the two-step approach, and Galleri across different cancer stages and types in the case-control study**

|  | **OncoSeek** | **Two-step** | **Galleri*** |
| --- | --- | --- | --- |
| **Specificity** | 91.0% | 99.3% | 99.5% |
| **Sensitivity** |  |  |  |
| **Stage** |  |  |  |
| I | 41.4% | 24.7% | 16.8% |
| II | 48.7% | 33.0% | 40.4% |
| III | 58.3% | 52.3% | 77.0% |
| IV | 57.7% | 54.0% | 90.1% |
| **Cancer type** |  |  |  |
| Pancreas | 88.2% | 64.7% | 83.7% |
| Bladder | 80.0% | 80.0% | 34.8% |
| Cervix | 75.0% | 37.5% | 80.0% |
| Liver/bile-duct | 68.8% | 59.4% | 93.5% |
| Colorectum | 64.5% | 46.8% | 82.0% |
| Lung | 60.5% | 41.9% | 74.8% |
| Gallbladder | 60.0% | 40.0% | 70.6% |
| Ovary | 60.0% | 60.0% | 83.1% |
| Stomach | 46.4% | 35.7% | 66.7% |
| Lymphoma | 32.2% | 28.8% | 56.3% |
| Breast | 30.8% | 25.0% | 30.5% |
| Leukemia | 23.3% | 16.7% | 20.0% |
| Esophagus | 20.0% | 10.0% | 85.0% |

*The sensitivity and specificity of Galleri were based on GRAIL’s CCGA study (Klein EA et al. Ann Oncol 2021;32(9):1167-77).
